# Patients with severe mental illness and the ethical challenges related to confidentiality during family involvement: A scoping review

**DOI:** 10.3389/fpubh.2022.960815

**Published:** 2023-01-12

**Authors:** Marit Helene Hem, Bert Molewijk, Bente Weimand, Reidar Pedersen

**Affiliations:** ^1^Norwegian University of Science and Technology (NTNU) Social Research, Trondheim, Norway; ^2^Faculty of Health Studies, VID Specialized University, Oslo, Norway; ^3^Centre for Medical Ethics, Faculty of Medicine, University of Oslo, Oslo, Norway; ^4^Department Ethics, Law and Humanities, Amsterdam University Medical Centre (UMC) and Vrije Universiteit, Amsterdam, Netherlands; ^5^University of South-Eastern Norway, Faculty of Health and Social Sciences, Drammen, Norway; ^6^Division Mental Health Services, Akershus University Hospital, Lørenskog, Norway

**Keywords:** confidentiality, ethics, ethical challenges, ethics analysis, family involvement, scoping review, severe mental illness

## Abstract

**Background:**

Despite evidence on the significant potential value of family involvement during the treatment of patients with severe mental illness, research has shown that family involvement is largely underused. The duty of confidentiality is reported to be a key barrier to family involvement. To develop more insight into this barrier, this scoping review focuses on the following question: What are the reported ethical challenges related to confidentiality when involving family in the treatment of patients with severe mental illness?

**Methods:**

A systematic search into primary studies was conducted using the following databases: Medline (Ovid), PsycINFO (Ovid), CINAHL (EBSCO), and Web of Science core collection (Clarivate). The PICO (Population, Intervention, Comparison, Outcome) scheme and qualitative content analysis were used to make the ethical challenges more explicit.

**Results:**

Twelve studies—both qualitative and quantitative—were included. We identified the following main categories of ethical challenges: (1) the best interest of family members vs. confidentiality, (2) the patient's best interest vs. the right to confidentiality, (3) patient trust and alliance as a reason not to involve the relatives or not to share information, and (4) using confidentiality as a smokescreen. We also identified several subcategories and illustrative and concrete examples of ethical challenges.

**Conclusions:**

Through a systematic examination, we discovered various types of ethical challenges related to confidentiality when involving the family in the treatment of patients with severe mental illness. However, research on these ethical challenges and the constituents of these challenges remains limited and often implicit. An ethical analysis will create knowledge which may facilitate a more balanced and nuanced approach to respecting the principle of confidentiality while also considering other moral principles. The duty of confidentiality does not always have to be a major barrier to family involvement; this insight and using this ethical analysis in the training of healthcare professionals may benefit the patient, the family, and the services.

## Introduction

While researchers (1) and international health authorities (2–6) widely recognize family involvement as a key ingredient in mental healthcare for patients with severe mental illness, the inclusion of family and next of kin is often lacking or inadequate (1, 7, 8).

Important and frequently reported barriers to family involvement for patients with severe mental illness include patients' concerns about privacy (9) and healthcare professionals' duty of confidentiality (1). Health professionals argue that the duty of confidentiality takes precedence over relatives' need for information and involvement, while relatives claim that health professionals use the duty of confidentiality to avoid true collaboration (ibid.). Though confidentiality is one of the oldest and most renowned healthcare duties, this duty must be balanced against other duties or modified due to equally legitimate (and conflicting) interests and principles. One example is when a patient wishes to keep information from next of kin, but the next of kin wishes to be involved, for example, to support the patient.

Family involvement can be beneficial for the patient, health service, and family members (10, 11). Family involvement can contribute to relapse prevention and reduced hospital stays (12–14), secure the patient's interests when they are unable to do so, and achieve common goals and a common understanding of collaboration. Being in a close relationship with people with severe mental illness can be both meaningful and rewarding but also causes stress that contributes to poorer physical, mental, and social health to caregivers (15). Being involved in treatment can reduce the stresses family members experience (16–18). For health services, it is important to receive information about the patient from those who know the patient well. Simultaneously, the health workers should include next of kin and support them so that they can continue being important resources (3).

Several challenges give rise to problems when establishing family involvement in the treatment and care for people with severe mental illness. One such challenge is a lack of resources to be able to meet patients' and carers' legitimate needs in terms of time, routines, and professional knowledge. Research has demonstrated that health professionals describe such conflicts as leading to ethical challenges and dilemmas due to conflicting needs (8). The fact that health professionals describe the dilemmas as ethical may be because they do not necessarily see a difference between legal, professional, and ethical dilemmas; this lack of distinction is further complicated by the fact that these categories may be interwoven. However, to better deal with ethical challenges related to the duty of confidentiality—that is, in situations where healthcare professionals are uncertain, in doubt, or in disagreement on how to comply with the duty of confidentiality—we need nuanced knowledge on this topic. To help the whole triadic relationship to better cope and deal with these challenges, detailed knowledge and understanding of the ethical challenges at hand, seem to be important. An *ethical* analysis may not only serve as a basis for a better understanding of the intrinsic ethical dimensions of the duty of confidentiality, it can also give insight into how to deal with these ethical challenges.

During a preliminary literature search, we did not find any synthesis or summary of this topic. Thus, to contribute to an overview of research on ethical challenges and dilemmas related to confidentiality regarding triadic collaboration, we have analyzed and summarized the breadth and depth of the research (19) on this topic.

### Aim

The aim of this scoping review is two fold:

1. To identify empirical research on ethical challenges related to confidentiality when involving family and next of kin during the treatment of patients with severe mental illness.
2. To review this research and make the ethical challenges more explicit and clear and therefore also more manageable.

### Research question

What are the ethical challenges related to confidentiality when involving family in the treatment of patients with severe mental illness?

## Methods

### Search strategy

First, the research question was translated into a PICO scheme (population, intervention, comparison, and outcome; see Table 1 below). The population consisted of patients suffering from severe mental illness (SMI) and their network/family/next-of-kin. Intervention was formulated as family work/intervention/collaboration/cooperation with carers. The comparison was not specified and could be any or none. The outcome was defined as ethical challenges regarding confidentiality.

**Table 1:** Overview of the theme, categories and subcategories from the analysis.

**Theme**	**Patients with severe mental illness and the ethical challenges related to confidentiality during family involvement**			
**Categories**	**The best interest of family members vs. confidentiality**	**The patient's best interest vs. the right to confidentiality**	**Patient trust and alliance as a reason not to involve the relatives or not to share information**	**Using confidentiality as a smokescreen**
Sub-categories	° Keeping family at a distance may have negative consequences for the family members themselves ° Healthcare professionals' duty of confidentiality vs. relatives' legal rights and need for information ° Family members feeling neglected and unappreciated ° Family members receiving sparse information leads to challenges and stigma ° Family members are facing problems in retrieving information ° The importance of information relevant to the support role of carers	° Does the duty of confidentiality overrule the need to involve family? ° When family could provide helpful information	° Maintaining trust and building a therapeutic alliance with the patient ° Supporting family members ° Clinical judgment as an argument in favor of maintaining confidentiality	° Hiding behind the principle of confidentiality ° Uncertainty, misunderstanding, or overdoing the principle of confidentiality

Table 1 population, intervention, comparison and outcome (PICO):

- Population: patients suffering from severe mental illness (SMI) and their network/family/next-of-kin.
- Intervention: family work/- intervention/- collaboration/ cooperation with carers.
- Comparison: not defined (“any or none”).
- Outcome: ethical challenges regarding confidentiality.

The second step was to build search components to develop our search strategy. We formulated three “search blocks”: (1) SMI, (2) family involvement, and (3) confidentiality (see additional file for a detailed description of our search strategy). As the next step, relevant search terms and synonyms were formulated and added to the blocks. The fourth step included selecting relevant information sources. An academic librarian (MØ) systematically searched databases covering health and psychology, including Medline (Ovid), PsycINFO (Ovid), CINAHL (EBSCO), and Web of Science core collection (Clarivate). The Boolean operator “OR” was used within each block, while “AND” was used to combine the three blocks in the search. The initial search was performed in February 2018. In July 2018, an additional search was performed in the same databases utilizing the search term “next-of-kin”, and this search yielded one additional result (20). We performed the final update search in September 2022. There were no limits on the publication date. The search consisted of several synonyms for severe mental illness, in combination with synonyms for family relations and different aspects of the ethical challenges described earlier. We searched using both database-specific subject headings and in the fields for title, abstract, and author keywords. See Appendix 1 for full details of all searches.

After the search was executed, the results were collected into a reference management tool (Endnote). Non-research articles (theoretical papers, reviews, overviews, and commentaries), articles which were not available in English, and articles not available in full text (and/or where a detailed abstract was not available) were excluded.

### Study selection

All titles and abstracts retrieved through the literature searches were screened to identify studies that potentially fulfilled the inclusion criteria for this review. To assess the articles, the authors were divided into three pairs. To ensure consistency, the first author was part of all three pairs (MHH + BM, MHH + RP, MHH + BW). If one reviewer assessed a reference as potentially relevant, the full-text article was ordered. Each full-text article was assessed independently by the authors for inclusion or exclusion. Reference lists from the retrieved full-text articles were individually reviewed and scrutinized to detect any additional articles that were not identified in the computerized literature search.

### Data extraction

We extracted data from all the included studies using a data extraction sheet developed for the study. To assess the included studies, we extracted the following information: full reference, research purpose/aim, study design, theoretical perspective, context/setting (country, patient group, services/family involvement), results (ethical challenges related to confidentiality), and study limitations.

See Appendix 2 – Data extraction sheet.

### Inclusion criteria

We considered all studies of ethical challenges related to confidentiality when involving family and next of kin during the treatment of patients with severe mental illness. We limited the search to studies focusing on the perspectives of patients and/or family members/next of kin and/or healthcare staff. Furthermore, criteria for inclusion were peer-reviewed primary studies or systematic reviews. There were no limitations regarding the study design. The abstract had to be available, and publication language was limited to English.

### Exclusion criteria

Exclusion criteria included papers dealing with (ethical) challenges other than confidentiality and papers not focusing on SMI.

### Analytical procedure

We were inspired by Graneheim and Lundman (21) descriptions of qualitative content analysis and their concepts of “manifest” (inductive approach/identify ethical and practical challenges) and “latent” (deductive approach/theoretical/ethical approach) analysis. A latent analysis approach was also a necessity in the analytical work since we found no studies where there was an explicit focus on reporting and analyzing the ethical challenges or the ethical dimension of the reported challenges.

## Results

**Figure 1:**
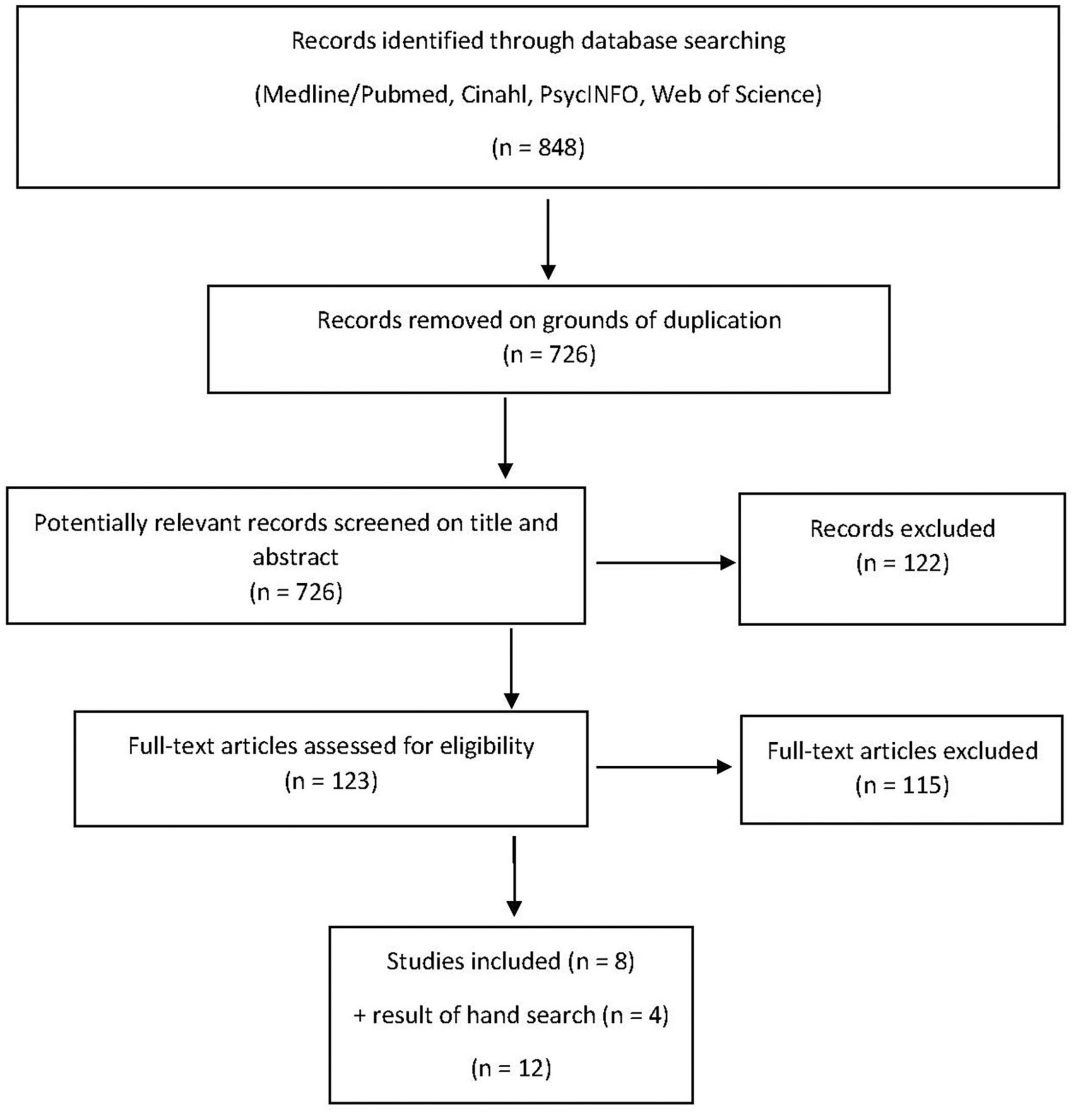
Study flow diagram with search strategy using PRISMA guidelines.

After extensive searches, 726 publications were identified for review following removal of duplicates with EndNote. Of these, eight research papers were included in this review. In addition, we identified seven articles by manual search, of which four were included (8, 22–24) (Figure 1).

### Ethical challenges related to confidentiality when involving family and next of kin

Based on our analyses of the selected papers, we found various types of ethical challenges related to confidentiality when involving family and next of kin during the treatment of patients with severe mental illness. We also learned that confidentiality related to different types of information resulted in different kinds of ethical challenges (e.g., information about the patient, the disease, or the treatment; information from next of kin for the healthcare professionals; or vice versa). Furthermore, the ethical challenges vary depending on stakeholder perspective (i.e., the caregiver, family, or patient perspective). In the following section, we present the results according to themes and subthemes (Table 1).

### The best interest of family members vs. confidentiality

#### Keeping family at a distance may have negative consequences for the family members themselves

McCann et al. [(25), p. 224], demonstrated that caregivers have contrasting experiences with mental health professionals. First, caregivers felt that most clinical staff were approachable and supportive. However, the study also determined that carers felt their support was undervalued by some health professionals when they were excluded from clinical considerations. Carers felt their role was not taken seriously by some health professionals. The carers' commitment to caring was affected by the way they interacted with health professionals both in first-episode psychosis service and on subsequent occasions. Cohesive, integrated, and supportive services contributed to carers' wellbeing as they struggled to provide support in light of their loved one's unpredictable behavior, anxiety, and worries as well as balancing the requirements of caregiving with other obligations.

Several of the studies found that there is poorer informal and voluntary care for the patient if the family does not receive information, support, and guidance. For example, Wilson et al. (24) observed that relatives' fighting with the mental health system leads to disempowerment as well as difficulty in accessing information, which is an argument against maintaining confidentiality. Similarly, Wainright et al. (26) highlighted that being kept out of the health and social care loop by professionals resulted in carers experiencing feelings of disempowerment, anger, loss, and isolation. Cree et al. (27) noted that:

Confidentiality was frequently raised as a barrier to carers becoming involved in both care planning and service users' care more generally. This was an emotive subject for many carers, and it could often reinforce self-blame for contributing to a service user's suffering. (ibid., p. 7)

Hence, sometimes patients' interests and the interests of relatives can both be affected at the same time by the strict application of confidentiality. Similarly, Wainwright et al. [(26), p. 110] found that relatives felt they were treated as non-essentials or even as rivals. Family members reported that they had to fight with services that were vague and ambiguous. The main areas of conflict when seeking help for their family member and for themselves included information about the patient and their care; confidentiality; neglect for family members' needs and wellbeing; lack of compassion; the structure of the services; and lack of information both general and specific to the patient. Per Wainwright et al. (26), “This lack of practical advice and information falls into two broad categories: General information about statutory and non-statutory services; and resources and information about the client and their care” (ibid., p. 110).

#### Healthcare professionals' duty of confidentiality vs. relatives' legal rights and need for information

Some papers clearly addressed reasons and/or practices that give more weight to the need to involve relatives. For example, an argument in favor of breaking confidentiality is the importance of recognizing relatives' legal right to receive information (8). Additionally, Rapaport et al. (23) discovered that there are few policies addressing information sharing with carers. Rapaport et al. (23) also presented examples of good practice in professionals' involvement of carers that embraced carer rights and responsibilities. This implies the importance of carers' right to an assessment of their circumstances and the care context as well as strengthening the argument for appropriate information sharing between professionals and carers (23), p. 357. In line with this, Førde et al. (20) maintained that acknowledging next-of-kin's role “as informal caregivers possessing valuable knowledge, does not threaten confidentiality” (p. 7). In Marshall and Solomon's (28) study, many family members described receiving information about diagnosis and medications, but few received information about the treatment plan. Family members were satisfied when they received information from healthcare practitioners (ibid.).

#### Family members feeling neglected and unappreciated

Førde et al. (20) showed that family members were involved in the patient's situation. However, they reported having negative experiences as family members in their meetings with mental healthcare professionals: “Not being seen and acknowledged as important caregivers and co-sufferers were experienced as offensive and could add to their feelings of guilt” (ibid., p. 1). Importantly, lack of involvement led to family members not sharing vital patient information with healthcare professionals. Family members reported that they felt neglected, unappreciated, and dismissed (20); this finding points to the importance of informing relatives to involve them as stakeholders within the social network of the patient. Similarly, according to Gray et al. (22), being rejected by professionals resulted in carers experiencing feelings of disempowerment, anger, loss, and isolation (p. 382).

Cree et al. (27) further echoes this finding:

Whilst identifying a shared desire for involvement and confirming a potential role for carers within services, our data highlighted that many carers perceive a lack of involvement in care planning and a lack of recognition and appreciation of their role from health professionals. (ibid., p. 1)

As a result, healthcare professionals may lose the potential value of carers taking advocacy roles in situations where patients themselves are less able—or less willing—to be involved in decisions regarding care. This may lead to a hierarchy in mental health care planning “in which the relative contributions of different stakeholders are determined more by role status than by their potential expertise” (ibid., p. 8). In today's mental healthcare, where values such as respect, equality, and autonomy are strong, such a practice is ethically problematic.

#### Family members receiving sparse information leads to challenges and stigma

According to Wainwright et al. (26), relatives felt that the information they received was disparate and sparse, leading to them not knowing what information might be lacking or what they should look for. They did not have any idea of “the pathway that a client might take on entering services, or for themselves, they cannot imagine what information might be helpful to them or where they might seek it” (p. 111). In addition, Gray et al. (22) remarked that the interests of the patient came first: the “rights and needs of carers for information sharing were a secondary concern” (p. 381). Furthermore, relatives felt that they were seen by professionals as the enemy. Some believed that information that might be important to their own welfare was suppressed. In addition, they described their own, sometimes traumatic, emotional difficulties:

They became responsible for managing risks; had to witness and manage challenging and bizarre behavior; and suffer the erosion of the hopes that they had for the future. They remark, however, on the apparent lack of empathy and understanding that services have for these experiences. [(26), p. 111]

To accommodate for and understand mental health crises, family members try to find explanations for and meaning in situations involving self-harm, violence, and detention. However, the studies determined that the mental health system does not address these problems. Family members also reported a lack of practical advice in coping with difficult behaviors, risks, financial problems, and so forth (ibid.). Family members sometimes stated that lack of acknowledgment, information, and support intensified feelings of guilt and harmed their ability to cope and care for their loved one. They explained that lack of information about the illness made it more difficult to inform others (e.g., bosses, the patient's job/school, friends, and family) about what was going on, which again may lead to stigma and lack of understanding and support (ibid.). Furthermore, Wainwright et al. (26) mentioned the problem of stigma:

Relatives believe that there is still a significant amount of stigma surrounding mental illness, despite anti-stigma campaigns. Not only is stigma aimed at service users, but relatives think that they too are stigmatized by professionals within the NHS [the healthcare system, our comment], including mental health professionals. (ibid., p. 112)

#### Family members are facing problems in retrieving information

In Wilson et al. (24), 56.3% of respondents reported that they faced difficulties retrieving information from mental health professionals. The main reasons for refusals to give information included lack of patient consent (46.2%) and unavailability of a team member (46.2%). Carers stated that the primary reason they felt there were problems in retrieving information was a lack of concern for their role as carer (60.5%). More than 75% of respondents were anxious about negative consequences for them or for the patient because of information being denied by mental health professionals (ibid., p. 781).

#### The importance of information relevant to the support role of carers

Some of the studies recorded challenges when opening up for a little less strict practice of the duty of confidentiality and more involvement of relatives. In the study by Slade et al. (29), relatives understood and respected the principle of confidentiality. Hence, there was no ethical conflict as such. Yet relatives experienced negative ethical consequences of following the principle of confidentiality:

Carers accepted the service user's right to withhold consent, but (like service users) acknowledged this might have an impact on the standard of care they can provide. They emphasized the importance of information relevant to their support role but, did not need or want to know everything about the person supported. [(29), p. 151]

Wainwright et al. [(26), p. 116] found that, over time, relatives learned to master the difficult situations that arise when supporting a family member who is experiencing psychosis. Families developed skills to cope with crises and prevent relapse, but they would rather be given suitable and proper information and support from a very early stage to learn these skills quickly. Indeed,

… carers spoke of the tensions that could emerge when a service user was acutely unwell and could become hostile toward the carer if they had been involved in the care planning. This became increasingly pertinent if compulsory measures had been used, particularly if these measures had been initiated by the carer, demonstrating the often competing challenges that are inherent in a carer's role. [(27), p. 7]

### The patient's best interest vs. the right to confidentiality

#### Does the duty of confidentiality overrule the need to involve family?

One core ethical challenge which was found in the selected papers is connected to healthcare professionals' respecting the duty of confidentiality regarding patient information. Most of the papers present findings in which healthcare professionals report believing that the duty of confidentiality often overrules the need to involve relatives. One argument in favor of maintaining confidentiality is that patients have a legal right to confidentiality which should be respected (27); this means that information about the patient should not be released to family members without the patient's consent. Furthermore, Slade et al. (29) observed that:

The service user interviews were dominated by one issue: the importance of patient confidentiality. All stressed how consent to disclose should be obtained before information is shared with carers. The requirement for consent was strongly linked to self-esteem, privacy, personal choice, independence, autonomy, general wellbeing and empowerment. (ibid., p. 151)

However, some studies discuss that the kinds and levels of information are important for how to deal with the choice to share the information. Slade et al. (29) make a distinction between general information, which can always be shared without consent, and personal information, which is often unknown to the carer and therefore where consent needs to be considered. Hence, the difference between general information and personal information is critical. However, Rapaport et al. (23) added a third important category, personal-sensitive, in addition to general and personal information:

… general information relates to information about mental health issues (e.g., rights, treatments, diagnoses, and services) and personal information relates to the service user's condition, treatment and care plan. The category of personal-sensitive information was identified as highly sensitive personal information relating to matters such as sexual orientation. [(23), p. 363]

Moran et al. (30) deemed disclosure to be problematic, unproductive, and harmful in the doctor-patient relationship. Some of the psychiatrists in their study feared that “giving a diagnosis might harm the establishment of a positive and trusting relationship with patients” [(30), p. 1373]. The study found that psychiatrists feared that diagnosis disclosure could lead to patients' losing hope for their health and consequently the danger of treatment drop-out. In addition, psychiatrists feared negative family responses toward the patient if the diagnosis was disclosed to them, worrying that family members may use the diagnosis against the patient. Finally, psychiatrists' difficulties with the task of disclosure were also connected to an emotional challenge and fear of personal safety involving the risk of provoking negative and sometimes aggressive responses toward them: “several psychiatrists anticipated hostile physical responses after sharing the diagnosis” (ibid., p. 1375). Furthermore, the psychiatrists did not address the point of psychoeducation involving family and patients and instead concentrated on supporting and caring for patients (ibid., p. 1375).

#### When family could provide helpful information

However, the absence of patient consent for collaboration with or involvement of family members in treatments represents an ethical dilemma related to confidentiality. Sometimes receiving information from family members presupposes sharing general information about the patient that may be perceived as sensitive by the patient, for example, that the patient is ill or receives health care if this is not already known by the family. Furthermore, even sharing general information may in some jurisdictions require consent from the patient, or this may be in a legal gray zone (8, 27, 28, 31). Not involving the family members when the patients refuse, may deprive the patient of important benefits of good family involvement during severe mental illness, for example since the family often needs information and guidance to provide their best informal support for the patient. For example, in Weimand et al. (8) ethical tensions “appeared regarding confidentiality vs. what was best for the patient” (ibid., p. 292). They concluded that healthcare professionals considered confidentiality an obstacle to sharing information with relatives (ibid., p. 292). Chen et al. (31) determined that sharing information with families in mental healthcare involves competing demands between the patient's right to confidentiality and the family's wish to know. Healthcare professionals therefore “find themselves walking a fine line between adhering to confidentiality guidelines and working for the clients' best interests” (ibid., p. 1556) by involving the family.

### Patient trust and alliance as a reason not to involve relatives or not to share information

#### Maintaining trust and building a therapeutic alliance with the patient

Maintaining trust and building a therapeutic alliance is mentioned as a reason for not giving information to or receiving information from relatives (28). Chen's study (31) indicated that healthcare practitioners worried about losing the patient's trust, especially when the patient had difficulties building trust in the first place.

To use the information provided by families when the client–family relationship turned adversarial, case managers depended on whether the family wished to be revealed as the source of information. If the family did not give the case manager permission to do so, the case manager might employ the “coming in the back door” strategy. However, if tensions existed between the case manager and the family, the case manager tended to check with the client about the information when the family continued to contact the case manager after the client had revoked permission [(31), p. 1561].

Similarly, Weimand et al. (8) found that nurses' responsibility was first and foremost to the patient: “confidentiality was tied to the trusting alliance between the nurse and patient” (p. 292). Sometimes, however, “nurses had concerns about breaking confidentiality and consequently jeopardizing the patient's trust” (ibid., p. 292). Building a therapeutic alliance is often described as putting the patient first when there are conflicts or when the patient is lacking trust, and building an alliance based on a trusting relationship is put before relatives' needs.

Chen (31) questioned whether there are good reasons for breaking confidentiality:

If a client did not have a legal guardian, case managers generally agreed that it was the client's decision that determined whether or to what extent the family could be involved in treatment, except in crisis situations, such as when the client might harm him- or herself or others. (ibid., p. 1559)

Crisis situations create ethical challenges because they raise the question of when there is a sufficient crisis and who should decide how to handle the crisis. Chen discussed the consequences of healthcare professionals having contact with and sharing information with families in the absence of client permission: “Case managers recognized that, by receiving information from families in the absence of client permission, they walked a fine line between compliance with and violation of confidentiality laws” (ibid., p. 1561). This posed an ethical dilemma when families provided helpful information for the patient's treatment.

#### Supporting family members

Significant focus on creating an alliance with the patient risks the danger that the family's trust in healthcare professionals is compromised and that the relationship between the patient and their family may worsen. Reasons for healthcare professionals supporting family members were:

… the context framing the nursing care, aspects of the actors, and relational concerns between them. Competing or contradictory demands were found within these premises. Two paths were identified concerning the nurses' support of relatives: seeing the relative in the shadow of the patient or as an individual person. [(8), p. 285]

In Cree et al. (27), confidentiality was treated as a two-way process, meaning that carers sometimes wanted their own thoughts around care to be kept confidential. Sometimes professionals respected this, but some carers reported:

… how difficult situations had arisen which had impacted negatively on the service user/carer relationship, when they felt health professionals had breached their own confidentiality, by telling service users things that they had specifically asked them not to. These different standards or foci of confidentiality for professionals reflect the relegation of the carer's role and prioritization of the service user within services. [(27), p. 7]

Many aspects regarding the doctor-patient relationship seem to pull in the direction of focusing on the patient and keeping relatives at a distance. Involving relatives creates additional challenges, even if the involvement of relatives may be best in the long run.

#### Clinical judgment as an argument in favor of maintaining confidentiality

According to Slade et al. (29), clinical judgment is essential to balancing conflicting ethical requirements in this field. Their study determined that clinical judgment is an argument in favor of maintaining confidentiality. The participants emphasized that “the core role of individual judgement, relationships built upon openness, knowledge and trust, and the process” (ibid., p. 150). Likewise, Moran et al. (30) stressed the importance of clinical judgment:

Their concern for the patient, and fear of the family abandoning the patient, led the psychiatrists' decision to share specific information with the family (e.g., recovery) and refrain from referring directly to the diagnosis itself—schizophrenia, as well as refraining to address the medication and potential side effects. (ibid., p. 1375)

Slade et al. (29) argument in favor of the importance of clinical judgment concentrates on the relationship (of trust) between the patient and the healthcare practitioners, whereas Moran et al. (30) focused on clinical judgments regarding the relationship between the patient and the family.

### Using confidentiality as a smokescreen

#### Hiding behind the principle of confidentiality

A particular type of ethical challenge emerged in the literature to describe healthcare professionals using the confidentiality principle as an argument in an inappropriate way: “Confidentiality is seen as a shield behind which services sometimes hide. This was perceived as a lazy fallback position that staff used, as opposed to attempting to work with service users and relatives to find areas of compromise and attempt to build bridges within families” [(26), p. 111]. The reason for this “shield” might be that health professionals did not know how to grapple with patients' needs and families' needs, and the confidentiality principle was used as a kind of smokescreen to keep patients and relatives at a distance. Additionally, Gray et al. (22) mentioned that professionals tended to hide behind confidentiality as “confidentiality smokescreens create a type of ‘wall of silence'. This negatively impacts upon carers' involvement with services, limits the information to which carers have access and adversely impacts upon the knowledge which carers require to provide care” (ibid., p. 381). Furthermore, they stated that:

… professionals may sometimes use confidentiality issues as a reason to withhold information from carers. Professionals did not take up the challenge of acting as an intermediary to promote discussion between carers and service users concerning issues of confidentiality and appropriate information sharing. In many cases, confidentiality smokescreens, poor information sharing, and lack of dialogue resulted in professionals not identifying people as carers. (ibid., p. 381)

Weimand et al. (8) found that “although the nurses acknowledged relatives' need for, and legal right to receive, general information, they focused on patient confidentiality when arguing for difficulties regarding sharing information with relatives” (p. 292). However, the nurses in Weimand et al. (8) study were also concerned that “acting illegally would result in negative consequences for themselves and therefore felt almost unable to help” (p. 290). This is an interesting argument in favor of maintaining confidentiality.

#### Uncertainty, misunderstanding, or overdoing the principle of confidentiality

Hidden or ulterior motives can be described as an ethical challenge, at least if they emerge partly due to moral distress or uncertainty, such as how to balance the interests of the patient with the interests of the relatives, or how to involve the relatives when the patient has severe relational challenges. However,

Although professionals were reticent about information sharing and sometimes tended to erect confidentiality smokescreens to withhold information from carers in everyday practice, … sharing information was vital in cases involving risk management, the safety of service users and carers and for carers in crisis … Some professionals are still uncertain how far they can involve carers, even in scenarios of risk, possible harm and safety. [(22), p. 383]

Another example of misunderstanding or inappropriate use of the principle of confidentiality is given by Førde et al. (20): “The NOK [next of kin, our comment] describe that confidentiality considerations are given as a reason for not talking to and involving the NOK, even when there is a high probability that the patient's competence to consent is lacking” (p. 7).

Cree et al. (27) noted the competing challenges of the carer role. Participants reported experiences where they thought that the idea of confidentiality had been misapplied to exclude carers from the patient's care; this was perceived as upsetting for those concerned and diminished the supposed importance of carers within mental health services. Cree et al. (27) understand these challenges as “lack of understanding of the nuances of confidentiality in practice, rather than a deliberate misuse” (p. 7).

## Discussion

In this paper, we performed a scoping review of *ethical* challenges related to confidentiality when involving family and next of kin during the treatment of patients with severe mental illness. With this review, we not only aim to contribute to research but also to clinical practice by means of offering insights into the core ethical challenges of confidentiality and how to deal with them.

The most important ethical challenge related to confidentiality when involving family and next of kin during the treatment of patients with severe mental illness is balancing the best interests of family members vs. the need to protect the patient's privacy. This ethical challenge is closely related to our second finding regarding balancing the patient's best interest vs. the right to confidentiality. Our findings demonstrate the importance of balancing the patient's and the family's trust at the same time. The alliance between patients and healthcare professionals is the most frequently reported reason for not involving family or not sharing information with them. Furthermore, we found that using confidentiality as a smokescreen—meaning hiding behind the principle of confidentiality—was not uncommon.

### Core reflections related to themes

#### The duty of confidentiality and respecting the patient's autonomy

Many of the papers in this scoping review highlighted that the duty of confidentiality is grounded in the importance of respecting the autonomy of the patient (8, 29). Sometimes, relatives do not want to be involved in the situation of their family member for various reasons, but most often the duty of confidentiality seems to go against relatives' interests and need to take part in the life of their family member. Furthermore, how much detailed and sensitive information the relatives actually need—and what they already know—should also be considered (22, 29, 30).

Our review highlights that the importance of the duty of confidentiality depends on the perspective of the specific stakeholder(s) at hand. It is not only the alliance between healthcare professionals and patients that is important for the patient's health. Sometimes it is also morally right to protect the patient from relatives and to help the patient to set boundaries. This is the case in situations where relatives may be intruding in various ways or may be critical and disrespectful toward the patient. Relatives may themselves struggle with mental health problems; it may therefore be in the relatives' interest to be less involved, for instance, if they are abused by the patient and/or are exhausted due to heavy burden over time (9). However, such difficult experiences are rarely a reason for no contact or involvement. Often relatives try to understand, make meaning, and help in a situation that is difficult to understand and cope with, especially when the patient's ability to share important knowledge is limited. Therefore, trust is important when it comes to handling the duty of confidentiality. Building a trusting therapeutic relationship between the patient and health professionals is important due to the vulnerability of the patient. Based on a trusting relationship, the bonds between all stakeholders in the triad are strengthened because communication, information and participation are improved, which is what Chen et al. (31) describe as “walking a fine line”.

#### Alliance and confidence

According to Rapaport et al. (23), there is a general lack of confidence among health professionals in sharing information with family members. However, if family members do not receive recognition and support in their role, they will likely be less helpful to the patient, and in the worst case they may be harmful by being more worried and trying to help a seriously ill patient without understanding of how to do so properly. Therefore, too much focus on building the alliance between the patient and health professionals can only lead to relatives' distrust in health professionals, thereby weakening the relationship between patient and relatives. Furthermore, the patient may have special needs both to be understood and not to feel betrayed (26). Our analysis thus shows that a lack of communication between professionals, the patient, and the family may be particularly problematic (ibid.). Independent of how much information healthcare professionals can or are allowed to share with family members, the needs and perspectives of family members should be given sufficient weight. In the end, both the alliance between family members and the patient as well as the alliance between family members and health professionals are important (10, 11, 24).

#### Stigma

During our analysis, the question emerged whether withholding information does more harm than good (30). If, for example, there are assumptions that relatives are unreasonable and have a negative impact on the patient (e.g., “bad parent”), this may lead healthcare professionals to take a detached approach to family members. Healthcare professionals need to be open and seek guidance to address and scrutinize (potential) prejudices and stigma toward relatives (26). There are, however, relatively few studies that emphasize the possible negative consequences for having no or highly limited contact with relatives (between family and health professionals and family and patient). For example, Qi et al. (32) shed light on vulnerable families concerned about the need for confidentiality, but their worry was connected to the feeling of stigma rather than to stakeholders within the family having different views and needs in relation to confidentiality. In this case, the patient's best interest seems to be compatible with the concerns of the whole family. In line with this, Førde et al. (20) maintained that acknowledging relatives' role “as informal caregivers possessing valuable knowledge, does not threaten confidentiality” (p. 7). Their findings suggested that silence can lead to mistrust and worry because the absence of patient consent raises dilemmas when families provide helpful information for the patient's treatment (28, 33); what should healthcare professionals do with the information? Should healthcare professionals or the family themselves inform the patient that the family provided this information? Does the patient's refusal to share information about themselves with family also imply that the family is not allowed to share their information?

#### Weighing principles and time

This scoping review indicates that, to a large degree, overly narrow and rigid adherence to the duty of confidentiality may go against the patient's best interest in the long run. For instance, when the patient is lacking the capacity to give consent, family members—as a rule—have the right to be informed; however, this does not always take place due to strict interpretation of the law by health professionals. Using confidentiality as a smokescreen may be based on an overly narrow interpretation of the law. Ultimately, this reasoning may lead to relatives having the same right to receive information as any stranger—unless the patient is committed or lacks the capacity to consent—even though they are important informal caregivers.

On the other hand, this review also demonstrates that sometimes health professionals break confidentiality to strengthen the patient's autonomy later in the treatment process. In other words, establishing a good relationship with family members now may be helpful and promote the patient's health in the future (34). Overall, the duty of confidentiality should, like the principle of autonomy, be weighed against other principles like the patient's best interest (e.g., informal caregivers who want the best for their family member) and justice (the family's best interest should also be considered).

### The value of an ethics-based approach to the concept of confidentiality

This scoping review had a specific angle when examining the challenges—both in research and clinical practice—related to confidentiality and family member involvement. From an ethics perspective within our analyses, we tried to understand and acknowledge the values and norms behind the reported challenges (35). We believe that before trying to “solve” reported challenges, it is important to understand the so-called moral constituents of the challenges. It becomes easier to develop creative strategies and balance the duty of confidentiality with other duties and principles when we have a nuanced understanding of the challenges. For example, if we better understand the moral conflict between following the duty of confidentiality related to the treatment of the patient and the need of the family to receive some information to better help the patient as carers, then we can start an inquiry into the question of whether it is possible to cater to both moral aims and to respect both at the same time. Moral conflicts or dilemmas are often presented in an abstract way and then seem unsolvable. However, if we more closely examine the concrete meaning of these principles in a specific situation, it sometimes becomes clear that respecting confidentiality does not mean that no information at all can be shared (as noted above): some information can perhaps be shared while still respecting a specific meaning of confidentiality. Or, if it appears impossible to find a balanced compromise between conflicting principles, then one can deliberate on the overarching question of what the moral limits of respecting confidentiality are. Moreover, how can we repair or deal with the possible harm that can be caused when (not) respecting confidentiality? One way of jointly developing creative means of dealing with the inherent harm that comes along with moral dilemmas within the triadic relationships is by establishing ethics reflection groups (35) or moral case deliberation (36). A more general conclusion from our scoping review is that these moral constituents of reported challenges did not receive much attention in the studies used.

Another ethics-based insight is that the negative consequences of maintaining confidentiality do not imply that the principle of confidentiality itself is morally wrong. Upholding moral principles or duties like confidentiality can cause some disadvantages; that is why they are called “principles” or “duties”. Moral principles are not meant to be ideal for every situation, and moral principles always co-exist with other principles. There are good reasons for maintaining the duty of confidentiality. When experiencing moral challenges related to confidentiality, it is important to consider its reasons and aims. Choosing not to do something simply because of the principle of confidentiality is not enough. Considering and explaining the reasons for and aims of confidentiality allows us to engage in true moral deliberation and exchange of arguments. Such deliberation can ultimately lead to the conclusion that one should perhaps breach confidentiality in a specific situation. Even if the involved stakeholders do not reach a joint consensus, research on ethics supports that after deliberation all stakeholders can better accept and understand the dissensus (37, 38). Hence, even if ethics does not always create moral solutions, it can promote sincere dialogue and constructive relationships (37, 39). Overall, understanding the moral constituents of moral challenges related to confidentiality and learning to deal with these will be helpful for healthcare professionals and the triadic relationship.

A final reflection that emerged from our ethical analyses of the studies is that confidentiality can—and perhaps should—be perceived as a relational concept or a relational moral principle since it is about the exchange of information between *all* involved stakeholders. In our scoping review, in which the triadic relationships consisted of the healthcare professional(s), the patient, and the family member(s), confidentiality played a role in six ways (i.e., from each position to the other two positions). For example, the healthcare professional can also experience moral challenges when receiving information from family members about the patient (e.g., not knowing what to do with this information or in which way they should inform the patient about the fact that they have received information from family members). Thus, dealing with moral challenges related to confidentiality in a dialogical way is even more important. Seeing confidentiality as a relational—or in this study, triadic—concept, implies that all involved stakeholders in the triadic relationship should be aware that each stakeholder can be confronted with moral challenges related to confidentiality.

### Recommendations for future research and clinical practice

#### Research

There is a need for more research on the topic of confidentiality. For example, we need to know more about how to keep family members' information confidential. We still do not know enough about cultural differences in preferences for respecting patients' confidentiality. Since we assume that the issue of stigma is relevant regarding confidentiality issues, we need more knowledge about how to reduce stigma and the shame following stigma.

We also need empirical research (development, innovation, evaluation, and implementation) into how we can help the different stakeholders in dealing with confidentiality and related ethical challenges. Through information about the concept and the rules, we may create a course, a module, or a specific ethics support tool (40, 41) about the ethical challenges of confidentiality and how to deal with them, including skills training for discussing confidentiality with the triad, such as methods for ethics support like ethics reflection groups and moral case deliberation (35, 39).

#### Clinical practice

More practical clarification is needed about what confidentiality does and does not entail for all perspectives within the triad, including the possible negative consequences of breaching confidentiality. The smokescreen phenomenon, or excessive application of the duty of confidentiality, can occur for various reasons such as lack of competence, experience, and routines for cooperation with relatives.

In addition, more attention is needed to distinguish between types of information and how these types do (not) relate to confidentiality. Types of information include, for instance, general information, specific information about the patient, sensitive information, and information that the family member probably already knows. The type of information shared can impact the degree to which confidentiality is broken. There will also be different degrees of negative consequences to not having contact with relatives. All these points must be balanced against each other. Contact with family members also depends on competence among staff members, routines they have, legislation, and medical record systems. Training and pilots are needed on how to handle the core dilemmas of confidentiality. Furthermore, healthcare professionals would also profit from training in dealing with contradictory principles and values, such as how to balance two conflicting values/principles; when it is allowed to break confidentiality; and, even more important, if one feels they must break confidentiality, how can they do this in a morally appropriate way (i.e., even if one cannot uphold the duty to confidentiality, can they still demonstrate care and respect to those who perhaps suffer as a result?).

### Strengths and limitations

The papers we included in our study did not clearly formulate what ethical challenges are, and it was therefore not always clear for us as researchers whether to interpret challenges, problems, and dilemmas as ethical challenges or not. In response, we have chosen to interpret what was framed as “challenges”, “problems”, and “dilemmas” into “ethical challenges” and “ethical problems”. This might be a strength since naming a challenge as an ethical challenge implies that the moral dimension can be better addressed, which might lead to more creative ways of respecting or compromising the underlying moral values. However, this translation may increase the risk of misinterpretation and bias. Another limitation is that we do not cover important issues like high-risk or emergency contexts where the ethical obligations to protect the patient and others are different from a competent refusal in a non-emergency situation. Furthermore, our point concerning the protection of the patient from family does not arise from the studies reviewed but is our own observation.

## Conclusion

This scoping review presented not only various types of ethical challenges related to confidentiality when involving the family in care for patients with severe mental illness, it also demonstrated that confidentially can be at stake in each of the six different relationships within the triad. Knowledge about the moral constituents and meaning of confidentiality challenges, both in research and in the clinical context, may facilitate a more balanced, nuanced, and creative approach to respecting the principle of confidentiality. Offering ethics support or developing a thematic ethics support tool on this subject might help in dealing with confidentiality in a better way. Overall, this scoping review revealed that the duty of confidentiality has multiple meanings and implications. Confidentiality does not always have to be a major barrier to family involvement. This insight, and its use in the future training of healthcare professionals as well as in research, may benefit patients, families, and the services.

## Author contributions

All co-authors designed and planned the review approach together. MHH coordinated the scoping review and performed the search together with academic librarian MØ after consulting with all authors about the content of the PICO scheme. RP cooperated with MØ on the updated search. MHH performed the first selection of the hits (screening the abstracts based on inclusion criteria), after which all authors participated in reading equal portions of the abstracts and discussed the inclusion of those studies among each other. Next, the selected articles were evaluated in full-text by MHH while BM and RP divided the articles into equal portions for full-text review. The included articles were analysed by MHH while BM, BW, and RP contributed to the analysis scheme by adding labels that were missing. MHH was the main person responsible for the first draft of the paper, after which BM, BW, and RP participated in supplementing and revising the manuscript. All authors gave final approval of the manuscript.
