# Supplementary material for: Patients with severe mental illness and the ethical challenges related to confidentiality during family involvement: A scoping review
Source: Front Public Health. 2023 Jan 12;10:960815. doi: 10.3389/fpubh.2022.960815 (PMC9877517; doi:10.3389/fpubh.2022.960815)
Supplement: Supplementary file 1 [file Table_1.docx]

# Appendix 1

## **Search strategy – February 2018**

**Ovid MEDLINE(R) Epub Ahead of Print, In-Process & Other Non-Indexed Citations, Ovid MEDLINE(R) Daily and Ovid MEDLINE(R) <1946 to Present>**

Date of search: 12.02.2018
Search result: 98 references

| 1 | exp affective disorders, psychotic/ or exp bipolar disorder/ or exp psychotic disorders/ or exp schizophrenia/ | 160596 |
| --- | --- | --- |
| 2 | (psychos*s or psychotic or schizo* or (bipolar adj disorder)).tw. | 181459 |
| 3 | ((severe or serious) adj (mental* or psychological or psychiatric) adj (disorder* or ill* or problem* or disease? or diagnos* or patient*)).tw. | 8793 |
| 4 | 1 or 2 or 3 | 235333 |
| 5 | exp Professional-Family Relations/ | 13574 |
| 6 | (((family or families or relatives or carer* or friend* or (social adj network) or (social adj support) or (informal adj care) or (triad* adj care*) or (famil* adj care) or (personal adj network)) adj3 (therapy or psychotherapy or psychoeducation or educat* or counseling or work or treatment or intervent* or intervene* or management or approach or involv* or inclu* or participati*)) or (holistic adj2 approach*) or (home adj treatment) or network or (family adj conference) or (open adj dialogue) or (care adj plans) or (joint adj crisis adj plan) or triang* or (trial adj interagency) or (resource adj group adj assertive adj community adj treatment) or (shared adj decision adj making)).tw. | 385611 |
| 7 | 5 or 6 | 397433 |
| 8 | exp confidentiality/ | 49175 |
| 9 | exp Privacy/ | 13674 |
| 10 | (privacy or privat* or privy or confidential* or (personal adj2 information) or (duty adj2 warn) or Disclosure or Nondisclosure or anonym*).tw. | 142909 |
| 11 | 8 or 9 or 10 | 184040 |
| 12 | 4 and 7 and 11 | 114 |
| 13 | limit 12 to english language | 98 |

Key: / = subject heading, Exp = exploded subject heading, tw = title, abstract, kf = author supplied keywords in Medline, ADJn = word distance of maximum n words.

**PsycINFO <1806 to February Week 1 2018> (Ovid)**Date of search: 12.02.2018.

Search result: 232 references

| 1 | exp Affective Disorders/ or exp Schizophrenia/ or exp Bipolar Disorder/ or exp Psychosis/ | 244877 |
| --- | --- | --- |
| 2 | schizoaffective disorder/ | 2912 |
| 3 | exp Schizoid Personality Disorder/ | 637 |
| 4 | exp "SCHIZOPHRENIA (DISORGANIZED TYPE)"/ | 181 |
| 5 | exp Schizophreniform Disorder/ | 340 |
| 6 | (psychos*s or psychotic or schizo* or (bipolar adj disorder)).tw. | 186171 |
| 7 | ((severe or serious) adj (mental* or psychological or psychiatric) adj (disorder* or ill* or problem* or disease? or diagnos* or patient*)).tw. | 11313 |
| 8 | 1 or 2 or 3 or 4 or 5 or 6 or 7 | 312760 |
| 9 | (((family or families or relatives or carer* or friend* or (social adj network) or (social adj support) or (informal adj care) or (triad* adj care*) or (famil* adj care) or (personal adj network)) adj3 (therapy or psychotherapy or psychoeducation or educat* or counseling or work or treatment or intervent* or intervene* or management or approach or involv* or inclu* or participati*)) or (holistic adj2 approach*) or (home adj treatment) or network or (family adj conference) or (open adj dialogue) or (care adj plans) or (joint adj crisis adj plan) or triang* or (trial adj interagency) or (resource adj group adj assertive adj community adj treatment) or (shared adj decision adj making)).tw. | 171538 |
| 10 | privacy/ | 2249 |
| 11 | (privacy or privat* or privy or confidential* or (personal adj2 information) or (duty adj2 warn) or Disclosure or Nondisclosure or anonym*).tw. | 89803 |
| 12 | 10 or 11 | 89861 |
| 13 | 8 and 9 and 12 | 247 |
| 14 | limit 13 to english language | 232 |

Key: / = subject heading, Exp = exploded subject heading, tw = title, abstract, kf = author supplied keywords in Medline, ADJn = word distance of maximum n words.

**CINAHL (EBSCO)**Date of search: 12.02.2018
Search result: 105 references

| **#** | **Query** | **Results** |
| --- | --- | --- |
| S1 | (MH "Affective Disorders, Psychotic+") | 5,335 |
| S2 | (MH "Bipolar Disorder+") | 5,045 |
| S3 | (MH "Psychotic Disorders+") | 65,353 |
| S4 | (MH "Schizophrenia+") OR (MH "Schizotypal Personality Disorder") | 11,172 |
| S5 | TI ( (psychos*s or psychotic or schizo* or (bipolar N1 disorder)) ) OR AB ( (psychos*s or psychotic or schizo* or (bipolar N1 disorder)) ) | 18,141 |
| S6 | TI ( ((severe or serious) N1 (mental* or psychological or psychiatric) N1 (disorder* or ill* or problem* or disease? or diagnos* or patient*)) ) OR AB ( ((severe or serious) N1 (mental* or psychological or psychiatric) N1 (disorder* or ill* or problem* or disease? or diagnos* or patient*)) ) | 4,112 |
| S7 | S1 OR S2 OR S3 OR S4 OR S5 OR S6 | 73,203 |
| S8 | (MH "Professional-Family Relations") | 12,266 |
| S9 | TI ( (((family or families or relatives or carer* or friend* or (social N1 network) or (social N1 support) or (informal N1 care) or (triad* N1 care*) or (famil* N1 care) or (personal N1 network)) N3 (therapy or psychotherapy or psychoeducation or educat* or counseling or work or treatment or intervent* or intervene* or management or approach or involv* or inclu* or participati*)) or (holistic N2 approach*) or (home N1 treatment) or network or (family N1 conference) or (open N1 dialogue) or (care N1 plans) or (joint N1 crisis N1 plan) or triang* or (trial N1 interagency) or (resource N1 group N1 assertive N1 community N1 treatment) or (shared N1 decision N1 making)) ) OR AB ( (((family or families or relatives or carer* or friend* or (social N1 network) or (social N1 support) or (informal N1 care) or (triad* N1 care*) or (famil* N1 care) or (personal N1 network)) N3 (therapy or psychotherapy or psychoeducation or educat* or counseling or work or treatment or intervent* or intervene* or management or approach or involv* or inclu* or participati*)) or (holistic N2 approach*) or (home N1 treatment) or network or (family N1 conference) or (open N1 dialogue) or (care N1 plans) or (joint N1 crisis N1 plan) or triang* or (trial N1 interagency) or (resource N1 group N1 assertive N1 community N1 treatment) or (shared N1 decision N1 making)) ) | 72,966 |
| S10 | S8 OR S9 | 83,375 |
| S11 | (MH "Privacy and Confidentiality+") | 15,253 |
| S12 | TI ( (privacy or privat* or privy or confidential* or (personal N2 information) or (duty N2 warn) or Disclosure or Nondisclosure or anonym*) ) OR AB ( (privacy or privat* or privy or confidential* or (personal N2 information) or (duty N2 warn) or Disclosure or Nondisclosure or anonym*) ) | 41,556 |
| S13 | S11 OR S12 | 51,097 |
| S14 | S7 AND S10 AND S13  Limiters - English Language | 105 |

MH=CINAHL subject headings, TI = search title, AB= search abstract, Nn= word distance of maximum n words.

**Web of Science**Date of search: 12.02.2018
Search result: 108 references

| # 7 | [108](http://apps.webofknowledge.com/summary.do?product=WOS&doc=1&qid=14&SID=D2GIhHiWeqotudvtYwB&search_mode=CombineSearches&update_back2search_link_param=yes) | #5 AND #4 AND #3  **Refined by:** **LANGUAGES:** (ENGLISH )  Indexes=SCI-EXPANDED, SSCI, A&HCI, CPCI-S, CPCI-SSH, ESCI Timespan=All years |
| --- | --- | --- |
| # 6 | [126](http://apps.webofknowledge.com/summary.do?product=WOS&doc=1&qid=12&SID=D2GIhHiWeqotudvtYwB&search_mode=CombineSearches&update_back2search_link_param=yes) | #5 AND #4 AND #3  Indexes=SCI-EXPANDED, SSCI, A&HCI, CPCI-S, CPCI-SSH, ESCI Timespan=All years |
| # 5 | [334,538](http://apps.webofknowledge.com/summary.do?product=WOS&doc=1&qid=10&SID=D2GIhHiWeqotudvtYwB&search_mode=AdvancedSearch&update_back2search_link_param=yes) | TS= (privacy or privat* or privy or confidential* or (personal NEAR2 information) or (duty NEAR2 warn) or Disclosure or Nondisclosure or anonym*)  Indexes=SCI-EXPANDED, SSCI, A&HCI, CPCI-S, CPCI-SSH, ESCI Timespan=All years |
| #4 | [1,894,440](http://apps.webofknowledge.com/summary.do?product=WOS&doc=1&qid=7&SID=D2GIhHiWeqotudvtYwB&search_mode=AdvancedSearch&update_back2search_link_param=yes) | TS= (((family or families or relatives or carer* or friend* or (social NEAR network) or (social NEAR support) or (informal NEAR care) or (triad* NEAR care*) or (famil* NEAR care) or (personal NEAR network)) NEAR3(therapy or psychotherapy or psychoeducation or educat* or counseling or work or treatment or intervent* or intervene* or management or approach or involv* or inclu* or participati*)) or (holistic NEAR2 approach*) or (home NEAR treatment) or network or (family NEAR conference) or (open NEAR dialogue) or (care NEAR plans) or (joint NEAR crisis NEAR plan) or triang* or (trial NEAR interagency) or (resource NEAR group NEAR assertive NEAR community NEAR treatment) or (shared NEAR decision NEAR making))  Indexes=SCI-EXPANDED, SSCI, A&HCI, CPCI-S, CPCI-SSH, ESCI Timespan=All years |
| # 3 | [292,167](http://apps.webofknowledge.com/summary.do?product=WOS&doc=1&qid=6&SID=D2GIhHiWeqotudvtYwB&search_mode=CombineSearches&update_back2search_link_param=yes) | #2 OR #1 |
| # 2 | [21,064](http://apps.webofknowledge.com/summary.do?product=WOS&doc=1&qid=5&SID=D2GIhHiWeqotudvtYwB&search_mode=AdvancedSearch&update_back2search_link_param=yes) | TS=((severe or serious) NEAR (mental* or psychological or psychiatric) NEAR (disorder* or ill* or problem* or disease? or diagnos* or patient*))  Indexes=SCI-EXPANDED, SSCI, A&HCI, CPCI-S, CPCI-SSH, ESCI Timespan=All years |
| # 1 | [279,426](http://apps.webofknowledge.com/summary.do?product=WOS&doc=1&qid=4&SID=D2GIhHiWeqotudvtYwB&search_mode=AdvancedSearch&update_back2search_link_param=yes) | TS= (psychos*s or psychotic or schizo* or (bipolar disorder))  Indexes=SCI-EXPANDED, SSCI, A&HCI, CPCI-S, CPCI-SSH, ESCI Timespan=All years |

Key: TS = topic, which includes title, abstract, author keywords and Web of Science Keywords Plus.

# **April 2021 Search update**

**Ovid MEDLINE(R) ALL, 1946 to April 27, 2021**

Date of search: April 28, 2021

Search result: 144 references

| 1 | exp affective disorders, psychotic/ or exp bipolar disorder/ or exp psychotic disorders/ or exp schizophrenia/ | 178673 |
| --- | --- | --- |
| 2 | (psychos*s or psychotic or schizo* or (bipolar adj disorder)).tw. | 210278 |
| 3 | ((severe or serious) adj (mental* or psychological or psychiatric) adj (disorder* or ill* or problem* or disease? or diagnos* or patient*)).tw. | 11798 |
| 4 | 1 or 2 or 3 | 268180 |
| 5 | exp Professional-Family Relations/ | 15114 |
| 6 | (((family or families or relatives or carer* or friend* or (social adj network) or (social adj support) or (informal adj care) or (triad* adj care*) or (famil* adj care) or (personal adj network)) adj3 (therapy or psychotherapy or psychoeducation or educat* or counseling or work or treatment or intervent* or intervene* or management or approach or involv* or inclu* or participati*)) or (holistic adj2 approach*) or (home adj treatment) or network or (family adj conference) or (open adj dialogue) or (care adj plans) or (joint adj crisis adj plan) or triang* or (trial adj interagency) or (resource adj group adj assertive adj community adj treatment) or (shared adj decision adj making)).tw. | 538051 |
| 7 | 5 or 6 | 551073 |
| 8 | exp confidentiality/ | 54027 |
| 9 | exp Privacy/ | 16013 |
| 10 | (privacy or privat* or privy or confidential* or (personal adj2 information) or (duty adj2 warn) or Disclosure or Nondisclosure or anonym*).tw. | 181847 |
| 11 | 8 or 9 or 10 | 226616 |
| 12 | 4 and 7 and 11 | 144 |

Key: / = subject heading, Exp = exploded subject heading, tw = title, abstract, kf = author supplied keywords in Medline, ADJn = word distance of maximum n words.

**APA PsycInfo <1806 to April Week 3 2021>**

Date of search: April 28, 2021

Search result: 289 references

| 1 | exp Affective Disorders/ or exp Schizophrenia/ or exp Bipolar Disorder/ or exp Psychosis/ | 280261 |
| --- | --- | --- |
| 2 | schizoaffective disorder/ | 3168 |
| 3 | exp Schizoid Personality Disorder/ | 661 |
| 4 | exp "SCHIZOPHRENIA (DISORGANIZED TYPE)"/ | 185 |
| 5 | exp Schizophreniform Disorder/ | 358 |
| 6 | (psychos*s or psychotic or schizo* or (bipolar adj disorder)).tw. | 205859 |
| 7 | ((severe or serious) adj (mental* or psychological or psychiatric) adj (disorder* or ill* or problem* or disease? or diagnos* or patient*)).tw. | 13724 |
| 8 | 1 or 2 or 3 or 4 or 5 or 6 or 7 | 354783 |
| 9 | (((family or families or relatives or carer* or friend* or (social adj network) or (social adj support) or (informal adj care) or (triad* adj care*) or (famil* adj care) or (personal adj network)) adj3 (therapy or psychotherapy or psychoeducation or educat* or counseling or work or treatment or intervent* or intervene* or management or approach or involv* or inclu* or participati*)) or (holistic adj2 approach*) or (home adj treatment) or network or (family adj conference) or (open adj dialogue) or (care adj plans) or (joint adj crisis adj plan) or triang* or (trial adj interagency) or (resource adj group adj assertive adj community adj treatment) or (shared adj decision adj making)).tw. | 205819 |
| 10 | privacy/ | 2820 |
| 11 | (privacy or privat* or privy or confidential* or (personal adj2 information) or (duty adj2 warn) or Disclosure or Nondisclosure or anonym*).tw. | 104601 |
| 12 | 10 or 11 | 104669 |
| 13 | 8 and 9 and 12 | 289 |

Key: / = subject heading, Exp = exploded subject heading, tw = title, abstract, kf = author supplied keywords in Medline, ADJn = word distance of maximum n words.

**CINAHL (EBSCO)**

Date of search: April 28, 2021

Search result: 179 references

| **#** | **Query** | **Results** |
| --- | --- | --- |
| S1 | (MH "Affective Disorders, Psychotic+") | 12,808 |
| S2 | (MH "Bipolar Disorder+") | 12,227 |
| S3 | (MH "Psychotic Disorders+") | 128,499 |
| S4 | (MH "Schizophrenia+") OR (MH "Schizotypal Personality Disorder") | 26,512 |
| S5 | TI ( (psychos*s or psychotic or schizo* or (bipolar N1 disorder)) ) OR AB ( (psychos*s or psychotic or schizo* or (bipolar N1 disorder)) ) | 49,862 |
| S6 | TI ( ((severe or serious) N1 (mental* or psychological or psychiatric) N1 (disorder* or ill* or problem* or disease? or diagnos* or patient*)) ) OR AB ( ((severe or serious) N1 (mental* or psychological or psychiatric) N1 (disorder* or ill* or problem* or disease? or diagnos* or patient*)) ) | 7,497 |
| S7 | S1 OR S2 OR S3 OR S4 OR S5 OR S6 | 148,095 |
| S8 | (MH "Professional-Family Relations") | 16,963 |
| S9 | TI ( (((family or families or relatives or carer* or friend* or (social N1 network) or (social N1 support) or (informal N1 care) or (triad* N1 care*) or (famil* N1 care) or (personal N1 network)) N3 (therapy or psychotherapy or psychoeducation or educat* or counseling or work or treatment or intervent* or intervene* or management or approach or involv* or inclu* or participati*)) or (holistic N2 approach*) or (home N1 treatment) or network or (family N1 conference) or (open N1 dialogue) or (care N1 plans) or (joint N1 crisis N1 plan) or triang* or (trial N1 interagency) or (resource N1 group N1 assertive N1 community N1 treatment) or (shared N1 decision N1 making)) ) OR AB ( (((family or families or relatives or carer* or friend* or (social N1 network) or (social N1 support) or (informal N1 care) or (triad* N1 care*) or (famil* N1 care) or (personal N1 network)) N3 (therapy or psychotherapy or psychoeducation or educat* or counseling or work or treatment or intervent* or intervene* or management or approach or involv* or inclu* or participati*)) or (holistic N2 approach*) or (home N1 treatment) or network or (family N1 conference) or (open N1 dialogue) or (care N1 plans) or (joint N1 crisis N1 plan) or triang* or (trial N1 interagency) or (resource N1 group N1 assertive N1 community N1 treatment) or (shared N1 decision N1 making)) ) | 160,739 |
| S10 | S8 OR S9 | 174,959 |
| S11 | (MH "Privacy and Confidentiality+") | 23,753 |
| S12 | TI ( (privacy or privat* or privy or confidential* or (personal N2 information) or (duty N2 warn) or Disclosure or Nondisclosure or anonym*) ) OR AB ( (privacy or privat* or privy or confidential* or (personal N2 information) or (duty N2 warn) or Disclosure or Nondisclosure or anonym*) ) | 84,459 |
| S13 | S11 OR S12 | 98,729 |
| S14 | S7 AND S10 AND S13 | 179 |

MH=CINAHL subject headings, TI = search title, AB= search abstract, Nn= word distance of maximum n words.

**Web of Science Core Collection: Citation Indexes**

Science Citation Index Expanded (SCI-EXPANDED) --1900-present, Social Sciences Citation Index (SSCI) --1900-present, Arts & Humanities Citation Index (A&HCI) --1975-present, Conference Proceedings Citation Index- Science (CPCI-S) --1990-present, Conference Proceedings Citation Index- Social Science & Humanities (CPCI-SSH) --1990-present, Emerging Sources Citation Index (ESCI) --2015-present

Data last updated: 2021-04-27

Date of search: April 28, 2021

Search result: 167 references

| #6 | 167 | #5  AND  #4  AND  #3  Indexes=SCI-EXPANDED, SSCI, A&HCI, CPCI-S, CPCI-SSH, ESCI Timespan=All years |
| --- | --- | --- |
| #5 | 475,342 | TS= (privacy or privat* or privy or confidential* or (personal NEAR2 information)  or  (duty NEAR2 warn)  or  Disclosure  or  Nondisclosure  or  anonym*)  Indexes=SCI-EXPANDED, SSCI, A&HCI, CPCI-S, CPCI-SSH, ESCI Timespan=All years |
| #4 | 2,604,108 | TS=  (((family or families or relatives or carer* or friend* or (social NEAR network)  or  (social NEAR support)  or  (informal NEAR care)  or  (triad* NEAR care*)  or  (famil* NEAR care)  or  (personal NEAR network) )  NEAR3(therapy  or  psychotherapy  or  psychoeducation  or  educat*  or  counseling  or  work  or  treatment  or  intervent*  or  intervene*  or  management  or  approach  or  involv*  or  inclu*  or  participati*))  or  (holistic NEAR2 approach*)  or  (home NEAR treatment)  or  network  or  (family NEAR conference)  or  (open NEAR dialogue)  or  (care NEAR plans)  or  (joint NEAR crisis NEAR plan)  or  triang*  or  (trial NEAR interagency)  or  (resource NEAR group NEAR assertive NEAR community NEAR treatment)  or  (shared NEAR decision NEAR making) )  Indexes=SCI-EXPANDED, SSCI, A&HCI, CPCI-S, CPCI-SSH, ESCI Timespan=All years |
| #3 | 341,526 | #2  OR  #1  Indexes=SCI-EXPANDED, SSCI, A&HCI, CPCI-S, CPCI-SSH, ESCI Timespan=All years |
| #2 | 27,493 | TS= ((severe  or  serious)  NEAR  (mental* or psychological or psychiatric)  NEAR  (disorder* or ill* or problem* or disease? or diagnos* or patient*) )  Indexes=SCI-EXPANDED, SSCI, A&HCI, CPCI-S, CPCI-SSH, ESCI Timespan=All years |
| #1 | 324,396 | TS= (psychos*s or psychotic or schizo* or (bipolar disorder) )  Indexes=SCI-EXPANDED, SSCI, A&HCI, CPCI-S, CPCI-SSH, ESCI Timespan=All years |

Key: TS = topic, which includes title, abstract, author keywords and Web of Science Keywords Plus

# **September 2022 - Search update**

Updated search strategy by academic librarian Marte Ødegaard

**Medline Ovid**

Ovid MEDLINE(R) ALL <1946 to September 08, 2022>. Advanced search.

Date of search: September 09^th^ 2022

165 references retrieved

| 1 | exp affective disorders, psychotic/ or exp bipolar disorder/ or exp psychotic disorders/ or exp schizophrenia/ | 189414 |
| --- | --- | --- |
| 2 | (psychos*s or psychotic or schizo* or (bipolar adj disorder)).tw. | 223307 |
| 3 | ((severe or serious) adj (mental* or psychological or psychiatric) adj (disorder* or ill* or problem* or disease? or diagnos* or patient*)).tw. | 13301 |
| 4 | 1 or 2 or 3 | 283034 |
| 5 | exp Professional-Family Relations/ | 15451 |
| 6 | (((family or families or relatives or carer* or friend* or (social adj network) or (social adj support) or (informal adj care) or (triad* adj care*) or (famil* adj care) or (personal adj network)) adj3 (therapy or psychotherapy or psychoeducation or educat* or counseling or work or treatment or intervent* or intervene* or management or approach or involv* or inclu* or participati*)) or (holistic adj2 approach*) or (home adj treatment) or network or (family adj conference) or (open adj dialogue) or (care adj plans) or (joint adj crisis adj plan) or triang* or (trial adj interagency) or (resource adj group adj assertive adj community adj treatment) or (shared adj decision adj making)).tw. | 632551 |
| 7 | (psychosocial adj (therapy or educat* or counseling or work or treatment or intervent*)).tw. | 9548 |
| 8 | or/5-7 | 654151 |
| 9 | exp confidentiality/ | 55969 |
| 10 | exp Privacy/ | 17200 |
| 11 | (privacy or privat* or privy or confidential* or (personal adj2 information) or (duty adj2 warn) or Disclosure or Nondisclosure or anonym*).tw. | 204084 |
| 12 | 9 or 10 or 11 | 249791 |
| 13 | 4 and 8 and 12 | 165 |

**PsycInfo Ovid**

APA PsycInfo <1806 to August Week 5 2022>. Advanced search.

Date of search: September 09^th^ 2022

350 references retrieved

| 1 | exp Affective Disorders/ or exp Schizophrenia/ or exp Bipolar Disorder/ or exp Psychosis/ | 298720 |
| --- | --- | --- |
| 2 | schizoaffective disorder/ | 3260 |
| 3 | exp Schizoid Personality Disorder/ | 668 |
| 4 | exp "SCHIZOPHRENIA (DISORGANIZED TYPE)"/ | 186 |
| 5 | exp Schizophreniform Disorder/ | 361 |
| 6 | (psychos*s or psychotic or schizo* or (bipolar adj disorder)).tw. | 213929 |
| 7 | ((severe or serious) adj (mental* or psychological or psychiatric) adj (disorder* or ill* or problem* or disease? or diagnos* or patient*)).tw. | 14871 |
| 8 | 1 or 2 or 3 or 4 or 5 or 6 or 7 | 375232 |
| 9 | (((family or families or relatives or carer* or friend* or (social adj network) or (social adj support) or (informal adj care) or (triad* adj care*) or (famil* adj care) or (personal adj network)) adj3 (therapy or psychotherapy or psychoeducation or educat* or counseling or work or treatment or intervent* or intervene* or management or approach or involv* or inclu* or participati*)) or (holistic adj2 approach*) or (home adj treatment) or network or (family adj conference) or (open adj dialogue) or (care adj plans) or (joint adj crisis adj plan) or triang* or (trial adj interagency) or (resource adj group adj assertive adj community adj treatment) or (shared adj decision adj making)).tw. | 223583 |
| 10 | (psychosocial adj (therapy or educat* or counseling or work or treatment or intervent*)).tw. | 10024 |
| 11 | or/9-10 | 232260 |
| 12 | privacy/ | 3339 |
| 13 | (privacy or privat* or privy or confidential* or (personal adj2 information) or (duty adj2 warn) or Disclosure or Nondisclosure or anonym*).tw. | 112371 |
| 14 | 12 or 13 | 112453 |
| 15 | 8 and 11 and 14 | 350 |

**CINAHL**

Search modes - Boolean/Phrase, Interface - EBSCOhost Research Databases, Search Screen - Advanced Search

Date of search: September 09^th^ 2022

200 references retrieved

| S1 | (MH "Affective Disorders, Psychotic+") | 13,774 |
| --- | --- | --- |
| S2 | (MH "Bipolar Disorder+") | 13,179 |
| S3 | (MH "Psychotic Disorders+") | 139,581 |
| S4 | (MH "Schizophrenia+") OR (MH "Schizotypal Personality Disorder") | 28,266 |
| S5 | TI ( (psychos*s or psychotic or schizo* or (bipolar N1 disorder)) ) OR AB ( (psychos*s or psychotic or schizo* or (bipolar N1 disorder)) ) | 54,593 |
| S6 | TI ( ((severe or serious) N1 (mental* or psychological or psychiatric) N1 (disorder* or ill* or problem* or disease? or diagnos* or patient*)) ) OR AB ( ((severe or serious) N1 (mental* or psychological or psychiatric) N1 (disorder* or ill* or problem* or disease? or diagnos* or patient*)) ) | 8,301 |
| S7 | S1 OR S2 OR S3 OR S4 OR S5 OR S6 | 161,366 |
| S8 | (MH "Professional-Family Relations") | 17,710 |
| S9 | TI ( (((family or families or relatives or carer* or friend* or (social N1 network) or (social N1 support) or (informal N1 care) or (triad* N1 care*) or (famil* N1 care) or (personal N1 network)) N3 (therapy or psychotherapy or psychoeducation or educat* or counseling or work or treatment or intervent* or intervene* or management or approach or involv* or inclu* or participati*)) or (holistic N2 approach*) or (home N1 treatment) or network or (family N1 conference) or (open N1 dialogue) or (care N1 plans) or (joint N1 crisis N1 plan) or triang* or (trial N1 interagency) or (resource N1 group N1 assertive N1 community N1 treatment) or (shared N1 decision N1 making)) ) OR AB ( (((family or families or relatives or carer* or friend* or (social N1 network) or (social N1 support) or (informal N1 care) or (triad* N1 care*) or (famil* N1 care) or (personal N1 network)) N3 (therapy or psychotherapy or psychoeducation or educat* or counseling or work or treatment or intervent* or intervene* or management or approach or involv* or inclu* or participati*)) or (holistic N2 approach*) or (home N1 treatment) or network or (family N1 conference) or (open N1 dialogue) or (care N1 plans) or (joint N1 crisis N1 plan) or triang* or (trial N1 interagency) or (resource N1 group N1 assertive N1 community N1 treatment) or (shared N1 decision N1 making)) ) OR ((TI psychosocial OR AB psychosocial) W1 ((TI therapy OR AB therapy) OR (TI educat* OR AB educat*) OR (TI counseling OR AB counseling) OR (TI work OR AB work) OR (TI treatment OR AB treatment) OR (TI intervent* OR AB intervent*))) | 197,347 |
| S10 | S8 OR S9 | 212,081 |
| S11 | (MH "Privacy and Confidentiality+") | 24,871 |
| S12 | TI ( (privacy or privat* or privy or confidential* or (personal N2 information) or (duty N2 warn) or Disclosure or Nondisclosure or anonym*) ) OR AB ( (privacy or privat* or privy or confidential* or (personal N2 information) or (duty N2 warn) or Disclosure or Nondisclosure or anonym*) ) | 94,785 |
| S13 | S11 OR S12 | 109,429 |
| S14 | S7 AND S10 AND S13 | 200 |

**Web of Science (Clarivate)**

Database: Web of Science Core Collection

Science Citation Index Expanded (SCI-EXPANDED) --1900-present, Social Sciences Citation Index (SSCI) --1900-present, Arts & Humanities Citation Index (A&HCI) --1975-present, Conference Proceedings Citation Index- Science (CPCI-S) --1990-present, Conference Proceedings Citation Index- Social Science & Humanities (CPCI-SSH) --1990-present, Emerging Sources Citation Index (ESCI) --2005-present

Date of search: September 09^th^ 2022

133 references retrieved

| 1 | 1: TS= (psychos*s or psychotic or schizo* or (bipolar disorder) ) | 347724 |
| --- | --- | --- |
| 2 | 2: TS=((severe or serious) NEAR (mental* or psychological or psychiatric) NEAR (disorder* or ill* or problem* or disease? or diagnos* or patient*) ) | 32588 |
| 3 | 3: #2 OR #1 | 368366 |
| 4 | 4: TS= (((family or families or relatives or carer* or friend* or (social NEAR network) or (social NEAR support) or (informal NEAR care) or (triad* NEAR care*) or (famil* NEAR care) or (personal NEAR network) ) NEAR3(therapy or psychotherapy or psychoeducation or educat* or counseling or work or treatment or intervent* or intervene* or management or approach or involv* or inclu* or participati*)) or (holistic NEAR2 approach*) or (home NEAR treatment) or network or (family NEAR conference) or (open NEAR dialogue) or (care NEAR plans) or (joint NEAR crisis NEAR plan) or triang* or (trial NEAR interagency) or (resource NEAR group NEAR assertive NEAR community NEAR treatment) or (shared NEAR decision NEAR making) ) OR (TI=(psychosocial NEAR/0 (therapy OR educat* OR counseling OR work OR treatment OR intervent* )) OR AB=(psychosocial NEAR/0 (therapy OR educat* OR counseling OR work OR treatment OR intervent* ))) | 2315355 |
| 5 | 5: TS= (privacy or privat* or privy or confidential* or (personal NEAR2 information) or (duty NEAR2 warn) or Disclosure or Nondisclosure or anonym*) | 583942 |
| 6 | 6: #5 AND #4 AND #3 | 133 |
