# Supplementary material for: Patients with severe mental illness and the ethical challenges related to confidentiality during family involvement: A scoping review
Source: Front Public Health. 2023 Jan 12;10:960815. doi: 10.3389/fpubh.2022.960815 (PMC9877517; doi:10.3389/fpubh.2022.960815)
Supplement: Supplementary file 2 [file Table_2.docx]

**Appendix 2 – Data extraction sheet (original version)**

| **Author/year/title** | **Research purpose/aim** | **Study design** | **Context** | | | **Theoretical context/perspective(s)** | **Main outcomes/results:**  **Moral challenges related to confidentiality** | **Study limitations** |
| --- | --- | --- | --- | --- | --- | --- | --- | --- |
|  |  |  | **Country** | **Patient group** | **Services/Family involvement** |  |  |  |
| Chen et al., 2008.  A fine line to walk: case managers’ perspectives on sharing information with families. | Examining how case managers in assertive community treatment (ACT) programs conceptualize family involvement and how they work with client families | Semistructured,one-on-one interviews with 24 case managers | US | SMI | Assertive community treatment (ACT) programs where ACT is known as community support programs  (CSPs) | No theoretical context or perspectives, merely a descriptive paper (about empirical data and about literature that is being summarized) | Case managers were worried about losing the client’s trust, especially when trust building had been a difficult issue for the client.  Case managers were concerned about both the legal and the professional consequences of their contact with families in the absence of client permission.  Case managers recognized that, by receiving information from families in the absence of client permission, they walked a fine line between compliance with and violation of confidentiality laws.  Case managers respect clients’ confidentiality and do not release information without client consent.  The absence of client consent posts an ethical dilemma when families provide helpful information for clients’ treatment. Case managers find themselves walking a fine line between adhering to confidentiality guidelines and working for the clients’ best interests. | The scope of the current study is limited to clients  who make their own legal decisions.  The study focused on information sharing  within the ACT settings.  Findings regarding the practices of information sharing with families might not be applicable to other types of mental health services. |
| Cree et al. 2015.  Carers’ experiences of involvement in care planning: a qualitative exploration of the facilitators and barriers to engagement with mental health services | Addressing the gap in existing literature by examining carers' experiences of mental health services and care planning, exploring barriers and facilitators | Qualitative design: focus groups and interviews | UK | SMI | People who cared for someone with a severe mental illness |  | Confidentiality was frequently raised as a barrier to carers becoming involved in both care planning and service users’ care more generally. This was an emotive subject for many carers, and it could often reinforce self-blame for contributing to a service user’s suffering.  Confidentiality had been misused  previously to exclude carers from the service user’s care, acknowledging  the service users’ right to confidentiality.  Confidentiality was seen as a two-way process; carers sometimes wanted their own discussions around care to be kept confidential. | Only the views of carers are presented in the current paper. |
| Førde et al. 2016.  Next of kin’s experiences of involvement during involuntary hospitalization and coercion | Explores next-of-kins’ views and experiences of involvement  during involuntary hospitalisation | Qualitative interviews and focus groups | Norway | SMI (adults and adolescents) | Services: (involuntary) hospital treatment  Family involvement |  | NOK had experienced lack of involvement or had negative experiences as NOK in their encounters with the health services.  Not being seen and acknowledged as important caregivers and co sufferers were experienced as offensive and could add to their feelings of guilt.  Lack of involvement had as a consequence that vital patient information which the NOK possessed was not shared with the patient’s therapists. | The NOK of adult patients were recruited from NOK organizations which may recruit the most active, but possibly also the most frustrated part of the NOK population.  Women dominate this group of participants. |
| Gray et al. 2008.  ‘Conﬁdentiality smokescreens’ and carers for people with mental health problems: the perspectives of professionals | Examines confidentiality smokescreens  that erect barriers to information sharing and limit the inclusion of carers.  What do professionals think are the key  issues, and the legal, ethical, resource and practice  dilemmas facing themselves and carers? | Qualitative:  65 participants (strategic staff in mental healthcare delivery) were interviewed | UK | People with mental health problems | Carers for people with mental health problems/ Directors and senior staff from the health, social care and voluntary sectors/represent the experiences of carers from the perspectives of professionals |  | Service users came first while the rights and needs of carers for information sharing were a secondary concern. Confidentiality smokescreens create a ‘wall of silence’. This negatively impacts  upon carers’ involvement with services, limits the information to which carers have access and adversely impacts upon the knowledge which carers require in  order to provide care.  Carers are not only told that they cannot have information because of confidentiality issues, but are also unable to communicate vital information that would give professionals a more wellrounded picture of the service user’s illness and the challenges of care.  Professionals receive poor guidance and minimal support on methods to discuss  confidentiality and information sharing with carers and service users (left alone with legal, ethical and practical dilemmas of confidentiality and disclosure of information. | The experiences of carers from the perspectives of professionals are presented. |
| Marshall and Solomon 2000.  Releasing information to families of persons with severe mental illness: a survey of NAMI members | The purpose of the study was to understand the types of information shared and the process by which information was released to families of persons with severe mental illness. | Self-administered surveys were distributed  at the 1998 annual convention  of the National Alliance for the  Mentally Ill (NAMI). An additional  mailing was sent to 50 NAMI state offices five months later. NAMI members  distributed the surveys during  support group and educational meetings and also included the survey in NAMI newsletters. | US | SMI | Family and consumer members of the National Alliance for the Mentally Ill (NAMI) | Implicit theoretical/normative and somehow ‘activistic-protest’ perspective: evidence demonstrates that sharing information has better consequences, so we should do it. | Providers may be less likely to discuss family involvement and less likely to ask for permission to release information to families of clients who express negative attitudes toward their family.  Families who have been excluded from the treatment process may be skeptical of building collaborative relationships with providers.  Confidentiality lies at the core of the therapeutic relationship. The therapeutic relationship is based on clients’ trust that providers will not disclose information without their consent. However, many mental health agencies do not have clear procedures for releasing information to families. In most states, providing even the most basic information about a client’s condition or treatment without the client’s consent is technically a breach of confidentiality statutes (personal communication, Ulan H, Sept 1998). Nonetheless, the consent forms in use in many mental health agencies may not be appropriate for obtaining consumers’ authorization to release information to their families.  Morally loaded questions: At what point or points in the process do families typically receive information? Are there gender differences associated with the way that providers and families interact? Are mental health programs designed so that it is possible to include families in treatment planning? Are providers compensated (either reimbursed or allotted time) for collaborating with families? | Study respondents were mostly  parents of adults with mental illness,  white, and well educated. Because  NAMI is a support and education organization,  NAMI members have more access to information about mental illness: more likely to  understand the mental health system.  Consequently, NAMI members are significantly more likely to report contacts with professionals than are other family members  (not generalizable to all consumers and family members). |
| McCann et al. 2011.  Primary caregivers’ satisfaction with clinicians’ response to them as informal carers of young people with first-episode psychosis: a qualitative study | Explore ﬁrst-time primary caregivers’ experience of the way mental health nurses and other mental health clinicians respond to them as carers of young people. | A qualitative interpretative design: semi-structured, audio-recorded interviews with twenty primary caregivers | Australia | First-episode psychosis | Primary caregivers |  | Two competing themes were identiﬁed in the data, highlighting caregivers’ contrasting experience with mental health nurses and other mental health clinicians. First, most clinical staff were approachable and supportive. Second, several carers felt their contribution was undervalued by some clinical staff. This was as a consequence of being excluded from clinical deliberations because of clinical staffs’ concerns and young people’s requests about maintaining conﬁdentiality regarding treatment (balancing confidentiality with the need to know), as well as carers feeling their role was not taken seriously by clinical staff. | Generalisability is not obtained from sample representativeness but from the themes that are applicable to other carers and FEP clinical contexts.  Recruitment through case managers also might have led to an uncharacteristic sample of engaged caregivers with less critical beliefs about clinicians.  Most carers were women, and this limits the ﬁndings mainly to this gender, as females might have different conceptualisations of clinicians than males. |
| Moran et al. 2014.  Psychiatrists’ challenges in considering disclosure of schizophrenia diagnosis in Israel | To explore psychiatrists’ experiences and consideration of schizophrenia diagnosis disclosure to patients and/or family members | In-depth interviews with 14 psychiatrists from hospital and community settings.  Phenomeno-logical framework to analyze the interviews | Israel | SMI | Family members | Transformation of information disclosure:  history points towards the shift of norms and values | Psychiatrists experienced disclosure as problematic, unproductive, and harmful. 10 themes of psychiatrist experiences and concerns were identified conceptualized under three domains: (a) characteristics of schizophrenia, (b) the doctor–patient/family relationship, and (c) psychiatrists’ difficulties with the disclosure task.  Not explicitly formulated as moral challenges, yet many normative issues are mentioned:  ‘Although doctors might still have a degree of freedom to decide *how much* information to give and *when to give* it, they can no longer presume that patients do not want to know simply because they have not asked’.  Empirical consequentialistic argument for disclosure: ‘it is generally argued that candid revelation of diagnosis has a beneficial impact on patients irrespective of the gravity of one’s condition. The sharing of a medical diagnosis (or diagnosis disclosure) is consistent with contemporary health paradigms and ethics.’  Other arguments for disclosure are mentioned: diagnosis disclosure represents a shift from a traditional  paternalistic approach to an era of shared decision making centered on open dialogue and patients’ rights | Small sample and its specific Israeli cultural context.  Focus on schizophrenia limits generalizability of the findings with regard to other psychiatric conditions |
| Rapaport et al. 2006  Carers and conﬁdentiality in mental health care: considering the role of the carer’s assessment: a study of service users’, carers’ and practitioners’ views | To identify good practice in professionals sharing information  with carers with a view to developing a synthesised  model.  Although the aim derives from the ethical  standpoint of professionals providing information, the  research also encompassed the aspect of carers sharing  information with professionals on the basis that information  sharing usually involves two-way communication.  Three stakeholder groups were central to the project: users of mental health services, carers, and practitioners working in the field of mental health | Multiple method study comprising a policy search, a survey of service users, carers and professionals, and stakeholder interviews and  group events.  The study used three approaches, implemented  sequentially to gather data from the key stakeholders.  The data-gathering methods were a questionnaire  specifically designed for service users (  *n* = 168), carers, (n= 525, including 29 young carers aged less than  18 years), professionals (  n= 212) and carer support  workers (n  = 89); semi-structured stakeholder interviews  (34); and group events comprising stakeholder  groups (1 of service users, 3 of carers, 1 of professionals)  and two workshops involving service users, carers and  professionals | UK | SMI | Services: see column 3 | Ethical, legal and policy framework | Divided into   - Policy findings - Survey findings - Interview findings - Group events   The study found few policies that addressed the principles  underpinning information sharing with carers. However, examples of good practice in professional involvement of carers that took account of carer rights and responsibilities emerged from the research. | Self-selective nature of the survey  Absence of triangulation to validate results |
| Slade et al 2007.  Best practice when service users do not consent to sharing information with carers | To inform clinical practice when service users withhold consent to share information with their carer.  Developing a framework for best clinical practice where service user consent for sharing information with their carer is withheld | Data presented here were collected as part of a UK study assessing mental health  information-sharing practices across the life  course, including children and adolescents, adults of working age and older people.  Data were synthesised from a consecutive policy review (n=91, a national survey (n=595) of current policy review, a national survey of current practice and individual qualitative interviews (n=24).  Multiple methods of data collection were used to allow triangulation. Synthesising  quantitative and qualitative methods is the right approach in an area characterised by a complex and often conflicting set of polarised beliefs from different groups:  service users, carers and staff | UK | Mental  health service users of which 21% had been compulsory admitted | Carers, users and professionals |  | Interviewees identified both governing principles and specific strategies to guide information-sharing. They emphasized the core role of individual judgement, relationships built upon openness, knowledge and trust, and the process  The service user interviews were dominated by one issue: the importance of patient confidentiality. All stressed how consent to disclose should be obtained before information is shared with carers. The requirement for consent was strongly linked to self-esteem, privacy, personal choice, independence, autonomy, general wellbeing and empowerment.  Carers accepted the service user’s right to withhold consent, but (like service users) acknowledged this might have an impact on the standard of care they can provide.  They emphasised the importance of information relevant to their support role, but did not need or want to know everything about the person supported. Carers viewed professionals as often lacking the confidence, empathy, skills, time and organizational backing to fulfil a carer support role alongside provision of health and social care treatment for the service user.  The perspectives of professionals on information-sharing were largely consistent with carers and service users in emphasising confidentiality; context of care (length of relationship, type of illness, stage of recovery, living arrangements, past history); mental capacity and consent; and establishing service user and carer confidence in professionals. In addition, professionals identified that they had a duty to assess risk, to avoid harm and to use professional discernment for decision-making | Self-selection of respondents (affecting representativeness/generalizability: few people from black/ minority ethnic backgrounds).  Difficulties in accessing policy documents. The identification of policy about carers was problematic given the lack of an agreed definition of a ‘carer’. |
| Wainwright et al. 2015.  What do relatives experience when supporting someone in early psychosis? | Explore relatives’ experiences of supporting or caring for a relative/family member experiencing first episode psychosis.  Develop understanding of how to help relatives | Four focus groups, a total of 23 participants, thematic analysis | UK | Early psychosis | Descriptive (no required family intervention) | Theoretical perspective: despite governmental priorities to support relatives and carers this, many relatives of people experiencing psychosis continue to feel unsupported by mental health services. This may be due to lack of funding, high caseloads for mental health professionals, or due to a lack of understanding of what relatives experience as a result of their family member’s psychosis | Four key themes reﬂecting relatives’ understanding and management of psychosis were identiﬁed:  ‘Psychosis from the relatives’ perspective’  ‘Relatives’ ﬁght with the mental health ‘system’ (the main areas of conﬂict identiﬁed: information about the client and their care; conﬁdentiality; neglect for relatives’ needs and welfare; lack of empathy; services’ structure and functions being obscure and unknowable; and lack of information, both general and speciﬁc (to the client); Conﬁdentiality is seen as a shield behind which services sometimes hide.  ‘Is anybody listening? Does anyone understand?’; and  ‘Relatives’ coping’.  This study has clear implications for improvement in how relatives are supported in the United Kingdom such as; clearer guidance for staff about conﬁdentiality, treating relatives as partners in care and providing better quality information for relatives.  The ﬁndings demonstrate that relatives learn over time to cope and manage difﬁcult situations, which arise as a result of supporting a relative who is experiencing psychosis. It seems that, regardless of whether a family receive an intervention, over time the families develop skills to manage crises and prevent relapse, however, they would rather be provided with adequate and appropriate information and support from a very early stage to learn these skills quickly.  Clearer guidance on conﬁdentiality, treating relatives as partners in care, and providing better quality information for relatives are key.  Services need to move away from simplistic rules about conﬁdentiality and formalize procedures to allow relatives and carers access to the information they need, without impeding service users’ privacy and rights.  Confidentiality is seen as a shield behind which services sometimes hide. This was perceived as a lazy fallback position that staff used, as opposed to attempting to work with service users and relatives to find areas of compromise and attempt to build bridges within families. | Qualitative methodology: not generalize experiences to all relatives.  Volunteering to take part in the study: relatives may have been looking for an opportunity to share their experiences because they were particularly salient, negative, or distressing, and therefore their experiences may be different to others.  Context: experiences within UK mental health services; may differ between countries, however, other studies suggest similar challenges internationally, suggesting these ﬁndings and recommendations will have broad relevance.  Relatives’ experiences can be better explored using individual interviews to gain diversity and in-depth accounts (in stead of focus groups).  No explicit focus on the ethical dilemmas related to confidentiality, but rich empirical findings relevant to understand and better deal with confidentiality issues. |
| Weimand et al 2013  Nurses’ dilemmas concerning support of relatives in mental health care | Describe conceptions of nurses in mental health care about supporting relatives of persons with severe mental illness | Qualitative, descriptive study: focus group  interviews with nurses from all levels of mental health care | Norway | SMI | Relatives of persons with severe mental illness | Phenomenographic study: phenomenography  has its basis in cognitive psychology and was chosen in order to describe various perceptions of the phenomenon  under study: the nurses’ conceptions about supporting relatives | Ethical tensions appeared regarding confidentiality versus what was best for the patient, versus trying to support suffering family members.  The nurses found that their responsibility was the patient (develop an alliance).  Additional premises for supporting  relatives were the context framing the nursing care, aspects of the actors, and relational concerns between them. Competing or contradictory demands were found within these premises.  Two paths were identified concerning the nurses’ support of relatives: seeing the relative in the shadow of the  patient or as an individual person.  Confidentiality was strongly pointed out as an obstacle to communicating with relatives about patient issues. Although recognizing relatives’ legal rights to receive information, the nurses mostly described confidentiality as overriding. They feared that acting illegally would result in negative consequences for  themselves and therefore felt almost unable to help.  Some had shared information with relatives without letting the patient know, although it meant breaking  confidentiality. This was justified as being beneficial to the patient in the long run, and/or that the benefits for the relative would compensate for the disadvantage for the patient. | The descriptive categories and conceptions are built on  experiences made in the ‘‘real world,’’ and in that meaning constitutes a complex and interconnected whole.  Consequently, some overlaps between them.  Transferability of the findings: nurses who were willing to share their experiences may differ from  others.  By participating in a group discussion, group members influence each other.  Focus group  discussions might be biased since divergent opinions may be difficult to address.  Focus group interviews  may reveal some issues and conceal others.  May be national differences in the organization of mental health services (may be influencing the  transferability of findings) |
| Wilson et al. 2015  Mental health professionals and information sharing: carer perspectives | To identify the difficulties encountered by carers in relation to obtaining information from mental health teams | Cross sectional: information was gathered using an 18-item self-report questionnaire  (Carer Well-being and Support Questionnaire for carers of people with mental health problems or dementia) | Ireland | Persons with mental illness  (dementia,  schizophrenia, bipolar disorder/manic depression, depression, anxiety,  other mental health problem) | Carers/family members of persons with mental illness who were affiliated with a support group |  | A majority have encountered difficulties when attempting to access information resulting in per­ceived negative consequences.  One of the main reasons given by the treating team for this is lack of patient consent.  The results of the study support previous research, showing that carers often feel that their role is undervalued by the medical profession and their needs are overlooked. Most carers are satisfied with the support they receive from the mental health service. | Low response rate – not generalizable.  Self-selection of respondents - may not be representative/may bias the results.  Respondents may have been members of more than one of the organisations involved and therefore received duplicate copies of the questionnaire. |
